# Infective Endocarditis After a Splenectomy: A Complex Association

**DOI:** 10.7759/cureus.69143

**Published:** 2024-09-10

**Authors:** Jimmy Saleh, Stephen Georgiou, Mersal Samimi, Asseel Al-Bayati, Brandon Woodbury

**Affiliations:** 1 Internal Medicine, University of California San Francisco, Fresno, USA; 2 Cardiology, University of California San Francisco, Fresno, USA; 3 Cardiology/ Clinical Cardiac Electrophysiology, University of California San Francisco, Fresno, USA

**Keywords:** cardiac implantable electronic device (cied), infective endocarditis, post-splenectomy, staph aureus, vegetation

## Abstract

Infective endocarditis is a potentially life-threatening condition that can have grave cardiac and neurologic complications. Recognizing risk factors, such as the presence of Gram-positive bacteremia and cardiac devices, has improved early recognition and management. The spleen plays an important role in the immune response, helping protect the body from infection from various bacteria. However, there is a paucity of literature regarding post-splenectomy patients with cardiac devices and the risk of endocarditis in this population. We present a case of infective endocarditis as a late sequela post-splenectomy in a 60-year-old patient with a history of a dual-chamber pacemaker. The patient was initially found to have bacteremia after splenectomy with repeat transesophageal echocardiogram confirming a tricuspid vegetation. The patient ultimately required pacemaker extraction and a prolonged course of intravenous cefazolin. The clinical course was complicated by a septic pulmonary embolus.

## Introduction

Infective endocarditis (IE) is defined as an infection of a native or prosthetic heart valve, the endocardial surface, or a cardiac implantable electronic device (CIED). Patients with CIED are at an increased risk of developing IE due to the introduction of foreign material into the normally sterile heart environment. In recent decades, Staphylococcus has overtaken Streptococcus as the most common cause of IE [1]. Infection with these organisms can be a devastating complication in those with cardiac devices such as permanent pacemakers or cardioverter-defibrillators. The morbidity and mortality associated with IE in cardiac device infection is high [2] and current management includes early complete removal of the device [3-7]. In addition to CIEDs, other risk factors that increase the risk of IE include Gram-positive bacteremia, particularly *Staphylococcus aureus*, diabetes mellitus, long-term hemodialysis, immunosuppression, and IV drug use [8]. 

The spleen is the body's largest secondary lymphoid organ and is a vital component of our immune system. Predominantly, the spleen functions as a filter, removing old and damaged particles, which include red blood cells and blood-borne microorganisms. In addition, it plays an important role in the production of antibodies. This is often appreciated in patients as a large reduction in IgM memory B cells post splenectomy [9]. The loss of these cells leads to a decreased ability to opsonize bacteria, which is especially important in the body’s defense against encapsulated bacteria such as *Streptococcus pneumoniae, Neisseria meningitidis, *and* Haemophilus influenzae type B*. Due to the reduced ability to generate a robust immune response against these bacteria, asplenic and hyposplenic patients are more vulnerable to fulminant infections by these organisms [10]. This is why appropriate medical management strategies post splenectomy are extremely important, including immunization with pneumococcal, *Haemophilus influenzae* type B, and meningococcal vaccines [11].

We present the case of a 60-year-old patient with a CIED who underwent splenectomy several weeks before developing native tricuspid valve endocarditis caused by methicillin-sensitive *Staphylococcus aureus* despite a prolonged course of prophylactic antibiotics. He had a complicated clinical course that included the development of septic pulmonary embolus.

## Case presentation

A 60-year-old male with a history of sick sinus syndrome (SSS) status post pacemaker, tophaceous gout on chronic steroids, and peptic ulcer disease presented to the emergency department for severe abdominal pain along with subjective fever, chills, and left ankle pain. Prior to arrival, the patient had taken three tablets of nitroglycerin sublingually, as well as one inch of nitro paste, with minimal relief. On presentation, he was tachycardic and febrile. Lab results were significant for a lactic acid of 4 mmol/L, a C-reactive protein of 304 mg/L, and a procalcitonin of 10 ng/mL (Table 1), concerning for a bacterial infectious process. Blood cultures were obtained, and he was started on broad-spectrum antibiotics empirically. An X-ray of his left ankle revealed a moderate effusion. A CT scan of the abdomen and pelvis revealed a small amount of free air in the upper abdomen consistent with a perforated viscus.

**Table 1:** Lab values and reference ranges

	Patient's value	Reference range	Unit
Lactic acid	4.0	0.5 - 2.2	mmol/L
C-reactive protein	304.4	<=3.0	mg/L
Procalcitonin	10.53	<=0.30	ng/mL

The patient was emergently taken to the operating room, and a patch repair of a perforated gastric ulcer was completed. However, after the ulcer was sutured closed, the patient continued to have serosanguinous drainage from the left upper quadrant and was found to have multiple superficial capsular tears. A hemostatic agent was applied but was ineffective. Due to uncontrolled splenic bleeding, a splenectomy was performed. He was continued on antibiotics postoperatively. However, initial blood cultures returned positive for methicillin-sensitive *Staphylococcus aureus* (MSSA). In addition, a left ankle arthrocentesis was done, which also returned positive for MSSA. The patient’s antibiotics were transitioned to cefazolin based on susceptibilities, and the patient underwent further workup including a transthoracic echocardiogram (TTE) and a transesophageal echocardiogram (TEE), both of which were negative for IE.

**Figure 1:**
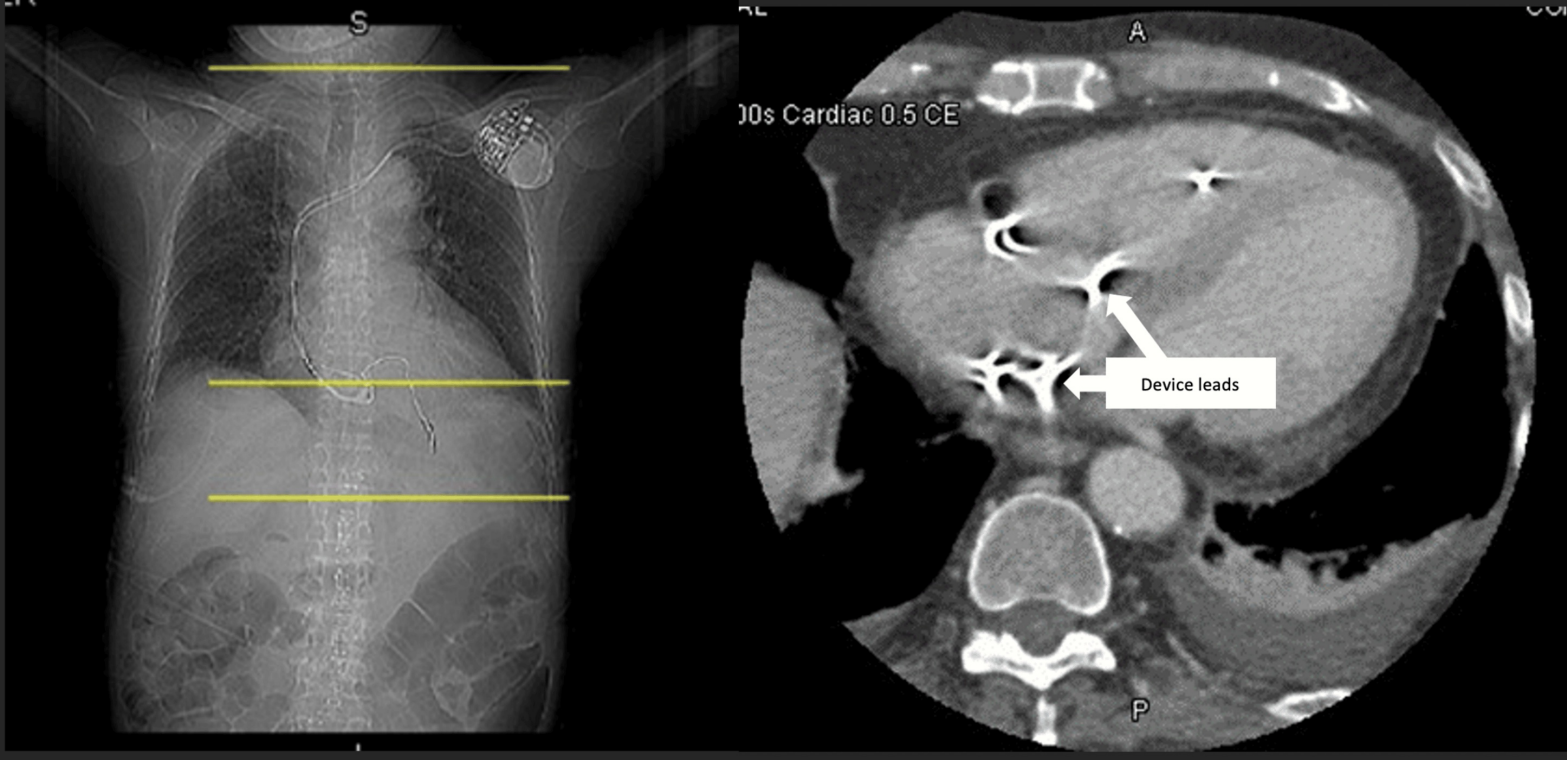
CT angio cardiac morphology 3D. Device leads seen as denoted by arrows. No specific abnormalities concerning for infection were seen.

After the initiation of antibiotics, repeat blood cultures were negative. He was evaluated by cardiology shortly after for pacemaker extraction. He underwent a CT angiogram cardiac morphology 3D with contrast (Figure 1) to evaluate the leads prior to extraction; it was unrevealing for any abnormality. The patient, however, developed recurrent leukocytosis despite being on cefazolin. Vancomycin was initiated and the infectious disease team was reconsulted. They evaluated the patient and recommended the removal of the pacemaker for the control of the MSSA bacteremia of unknown origin. A discussion with the patient regarding risks and benefits was held, and the patient elected to proceed with pacemaker removal.

**Figure 2:**
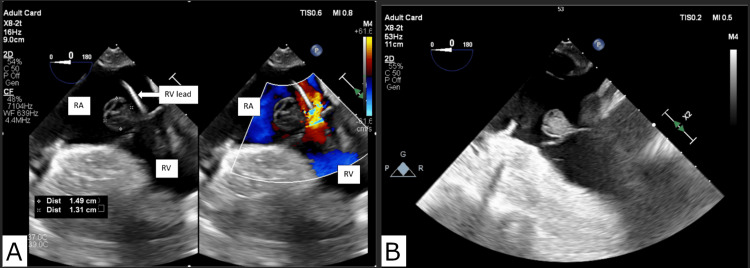
Intraoperative transesophageal echocardiogram (TEE). A) Vegetation prior to pacemaker lead extraction and B) after pacemaker lead extraction. RA: right atrium; RV: right ventricle

On the day of a scheduled pacemaker extraction, the patient developed septic shock. His antibiotics were broadened from cefazolin to vancomycin and Zosyn, and his pacemaker extraction was postponed for three days due to hemodynamic instability. Eventually, the patient was optimized for the procedure and underwent extraction of the pacemaker and leads. During the procedure, an intra-operative TEE revealed a tricuspid vegetation measuring 1.5 cm (Figure 2).

**Figure 3:**
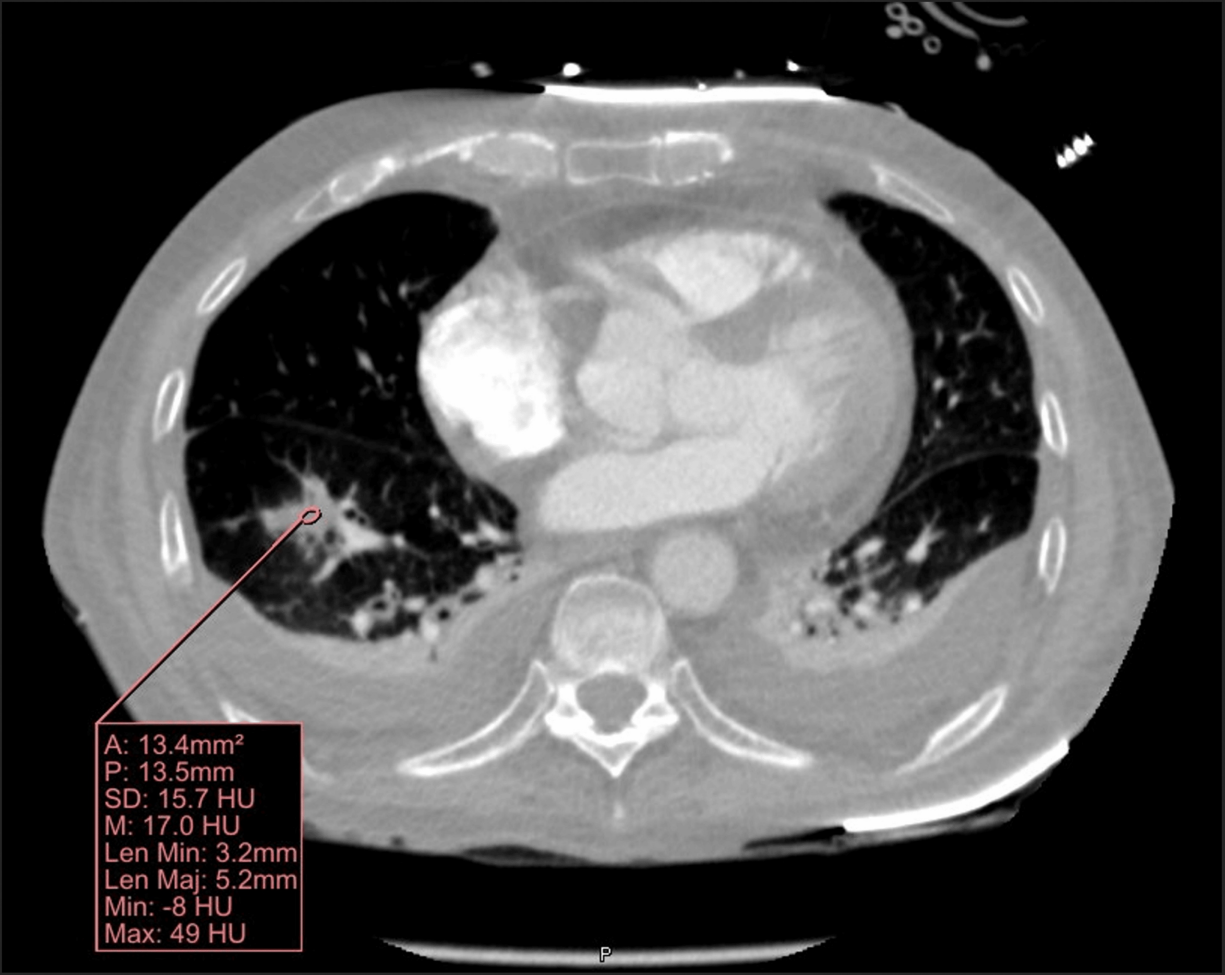
CT angiogram demonstrating a new septic pulmonary embolism.

**Figure 4:**
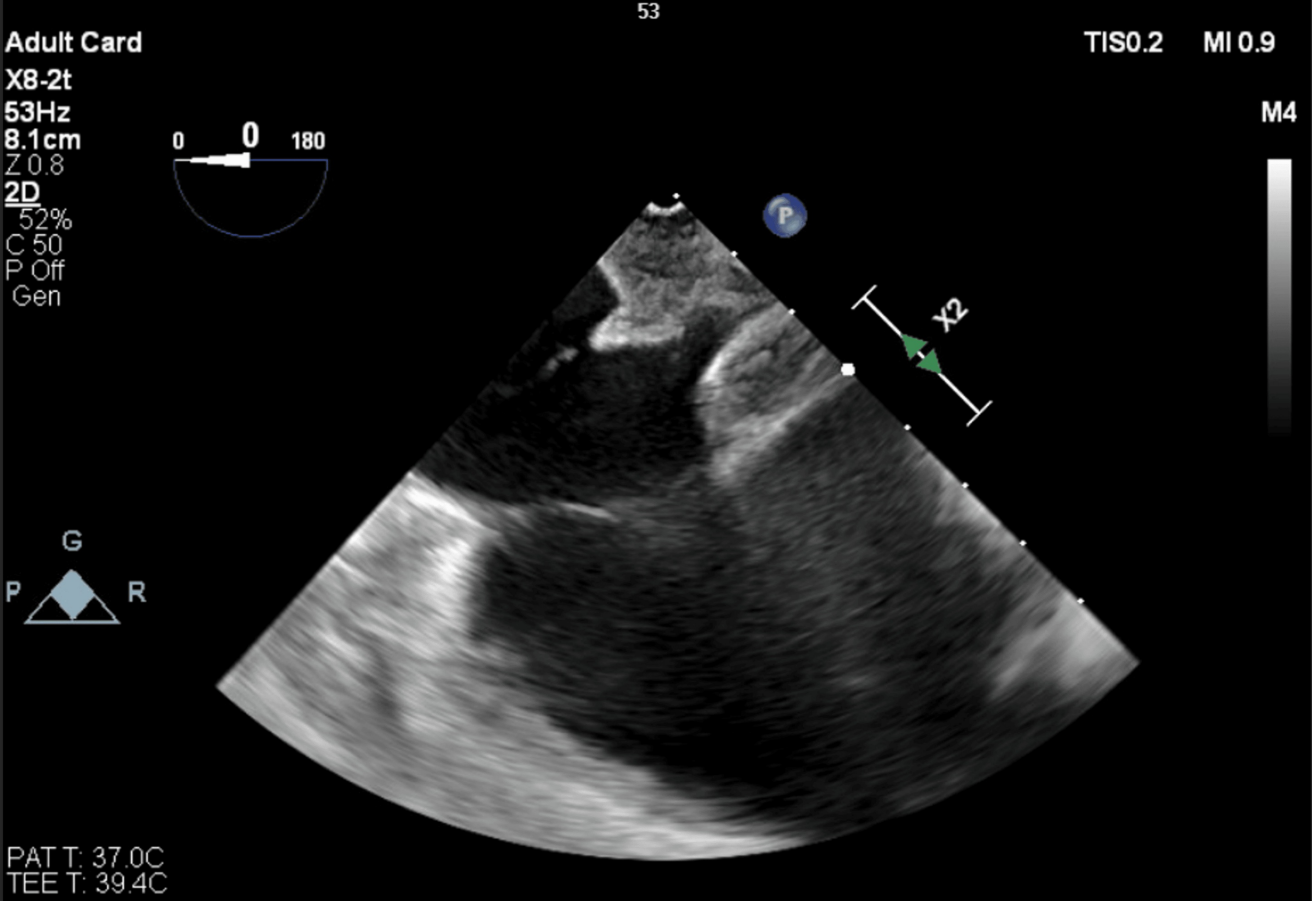
Repeat transesophageal echocardiogram (TEE) performed after the patient was found with a new pulmonary embolism, which no longer demonstrated previously visualized tricuspid valve vegetation.

Later in the day, after the procedure, the patient developed acute respiratory distress, and his clinical condition deteriorated. He was subsequently intubated and transferred to the intensive care unit. A CT angiogram was obtained, which revealed an acute pulmonary embolism of around 1.35 cm in size thought to be secondary to septic embolus (Figure 3). A follow-up TEE was obtained the same day, which no longer demonstrated the previously visualized tricuspid valve vegetation (Figure 4). The patient was continued on cefazolin for six weeks after a discussion between the infectious disease, cardiology, and cardiothoracic services. The patient was doing well until he developed a worsening leukocytosis four weeks into treatment with IV cefazolin. The treatment was broadened to IV cefepime. Repeat TTE one month later was unremarkable, and blood cultures remained negative. He finished his antibiotic course with IV cefepime after two additional weeks and was discharged.

## Discussion

IE is a serious and potentially life-threatening infection of the endocardium, often involving the valves of the heart. While splenectomy is a well-known cause of various medical conditions, such as increased susceptibility to certain infections, the association between splenectomy and an increased risk of IE is unknown. A previous case report exists highlighting the presence of native valve coagulase-negative endocarditis in a post-splenectomy patient [12]; however, the appearance of endocarditis as a late sequela despite targeted antibiotic therapy as seen in our case is unique.

The spleen plays a pivotal role in the immune system, particularly in filtering and clearing bacteria from the bloodstream. Splenectomy results in the loss of this vital function, rendering individuals more susceptible to certain infections, particularly those caused by encapsulated bacteria such as *Streptococcus pneumoniae, Haemophilus influenzae, Neisseria meningitidis,* and rarely *Staphylococcus aureus *[13]. This increased susceptibility to certain bacteria caused by the immunologic alterations caused by splenectomy could lead to a patient becoming more prone to developing infections, which in turn could cause a higher incidence of bacteremia as well as endocarditis. In addition, patients who have undergone splenectomy may present with atypical features of IE. The absence of the spleen as a primary line of defense can lead to a more aggressive course of infection, increased complications, and a higher risk of embolic events [14]. Clinicians should maintain a high index of suspicion for IE in these individuals, especially when encountering unexplained fever, murmur, or embolic phenomena. Per guidelines, early surgery and removal of implanted cardiac devices are recommended with evidence of device infection (Class I) and even without evidence of device infection when clinical suspicion is high (Class IIa) [15].

These guidelines, such as the updated statement on CIED infections published in Circulation [15], have also looked into various management techniques to decrease the incidence, as well as the mortality, of patients with CIED who develop IE. This includes the employment of pre-procedural antibiotics, predominantly first-generation cephalosporins such as cefazolin, to reduce the incidence of IE. The utility of antibiotic-eluting envelope placement or prophylactic treatment with vancomycin has also been debated. However, there remain no specific guidelines on the management of an asplenic patient with a CIED and their risk for endocarditis. For patients with a history of sepsis or severe infections in general that were caused by encapsulated organisms, such as *S. pneumoniae*, *H. influenzae*, *N. meningitidis*, or *S. aureus*, lifelong antibiotic prophylaxis can be considered [16-17]. However, further research elucidating the best clinical practice for this subset of patients, be it standardization of lifelong antibiotic prophylaxis or additional prophylactic measures in tandem with the currently established vaccination regimen against the most common encapsulated organisms, should improve patient outcomes and decrease patient mortality.

This case report, while providing another valuable example of the complexity of presentations in this specific patient population, does have its limitations. As with any case report, this patient’s presentation was unique, with the development of bacteremia despite being on antibiotic therapy, as well as having his course complicated by the development of a tricuspid vegetation that ultimately became a septic pulmonary embolus. Not all patients will have similar complications, and as such each patient's clinical course and management can vary, affecting the generalizability of this report. However, the complications our patient experienced further emphasize the importance of conducting more research on this topic, and ideally future studies will help elucidate improved strategies for prevention and management in this high-risk population.

## Conclusions

IE is a potentially life-threatening condition. Infections can become especially problematic in those with a weakened immune system. Our case report highlights a case of IE that developed in a patient who had undergone a previous splenectomy. The patient developed this infection despite being on appropriate antibiotics, and his clinic course was complicated by a tricuspid vegetation that ultimately became a septic pulmonary embolus. Patients who have undergone splenectomy are often at increased risk of infections, in particular by encapsulated organisms such as *S. pneumoniae*, *H. influenzae*, *N. meningitidis*, and, as in our patient, methicillin-sensitive *S. aureus*. Clinicians must be vigilant in recognizing the increased risk these individuals inherently have and implement preventive strategies to mitigate their potential complications. Further research is needed to refine guidelines for the management and prophylaxis of IE in patients with a history of splenectomy and CIED, with the ultimate goal of improving patient outcomes in this vulnerable population.

## References

[1] Cahill TJ, Prendergast BD (2016). Infective endocarditis. Lancet.

[2] Arber N, Pras E, Copperman Y (1994). Pacemaker endocarditis. Report of 44 cases and review of the literature. Medicine (Baltimore).

[3] Pfeiffer D, Jung W, Fehske W, Korte T, Manz M, Moosdorf R, Lüderitz B (1994). Complications of pacemaker-defibrillator devices: diagnosis and management. Am Heart J.

[4] Chua JD, Wilkoff BL, Lee I, Juratli N, Longworth DL, Gordon SM (2000). Diagnosis and management of infections involving implantable electrophysiologic cardiac devices. Ann Intern Med.

[5] Chamis AL, Peterson GE, Cabell CH (2001). Staphylococcus aureus bacteremia in patients with permanent pacemakers or implantable cardioverter-defibrillators. Circulation.

[6] Klug D, Lacroix D, Savoye C (1997). Systemic infection related to endocarditis on pacemaker leads: clinical presentation and management. Circulation.

[7] O'Nunain S, Perez I, Roelke M (1997). The treatment of patients with infected implantable cardioverter-defibrillator systems. J Thorac Cardiovasc Surg.

[8] Mylonakis E, Calderwood SB (2001). Infective endocarditis in adults. N Engl J Med.

[9] Kruetzmann S, Rosado MM, Weber H (2003). Human immunoglobulin M memory B cells controlling Streptococcus pneumoniae infections are generated in the spleen. J Exp Med.

[10] Cullingford GL, Watkins DN, Watts AD, Mallon DF (1991). Severe late postsplenectomy infection. Br J Surg.

[11] Di Sabatino A, Carsetti R, Corazza GR (2011). Post-splenectomy and hyposplenic states. Lancet.

[12] Abdi IA, Nur AA, Karataş M, Mohamud MF (2022). Post-splenectomy native valve endocarditis caused by coagulase negative staphylococci: a rare case report. Ann Med Surg (Lond).

[13] Ellison AM, Ota KV, McGowan KL, Smith-Whitley K (2013). Epidemiology of bloodstream infections in children with sickle cell disease. Pediatr Infect Dis J.

[14] Luu S, Spelman D, Woolley IJ (2019). Post-splenectomy sepsis: preventative strategies, challenges, and solutions. Infect Drug Resist.

[15] Baddour LM, Esquer Garrigos Z, Rizwan Sohail M (2024). Update on cardiovascular implantable electronic device infections and their prevention, diagnosis, and management: a scientific statement from the American Heart Association: endorsed by the International Society for Cardiovascular Infectious Diseases. Circulation.

[16] Caroline Hallam (2024). Splenectomy guidelines - patients with an absent or dysfunctional spleen CA4012 v6. NHS.

[17] Lee GM (2020). Preventing infections in children and adults with asplenia. Hematology Am Soc Hematol Educ Program.

